# Associated Factors with Perceived Fear of COVID-19 among Vietnamese Hospital Healthcare Workers during Fourth Wave of the COVID-19 Pandemic: Policy Implications for Interconnected and Social- and Personal-Based Health Support

**DOI:** 10.3390/healthcare9121713

**Published:** 2021-12-10

**Authors:** Quoc-Hung Doan, Nguyen-Ngoc Tran, Manh-Hung Than, Hoang-Thanh Nguyen, Van-San Bui, Dinh-Hung Nguyen, Hoang-Long Vo, Trong-Thien Do, Ngoc-Thach Pham, Tuan-Khanh Nguyen, Duc-Chinh Cao, Vu-Trung Nguyen, Thi-Mai T. Tran, Ba-Hien Pham, Anh-Long Tran, Van-Thuong Nguyen, Van-Thanh Nguyen, Xuan-Thang Tran, Thi-Lan Nguyen, Duc-Truong Lai, Quang-Hieu Vu, Satoko Otsu

**Affiliations:** 1 Department of Surgery, Hanoi Medical University, Hanoi 100000, Vietnam; hung.doanquoc@hmu.edu.vn; 2 Department of Cardiovascular and Thoracic Surgery, Viet Duc University Hospital, Hanoi 100000, Vietnam; 3 Hanoi Medical University Hospital, Hanoi Medical University, Hanoi 100000, Vietnam; buivansan@hmu.edu.vn; 4 Department of Psychiatry, Hanoi Medical University, Hanoi 100000, Vietnam; dotrongthien1794@gmail.com; 5 National Institute of Mental Health, Bach Mai Hospital, Hanoi 100000, Vietnam; 6 Emergency Department, National Hospital of Tropical Diseases, Hanoi 100000, Vietnam; 7 Office of Postgraduate Management, Hanoi Medical University, Hanoi 100000, Vietnam; Nguyenhoangthanh@hmu.edu.vn; 8 Hanoi Department of Health, Hanoi 100000, Vietnam; ndhung71@gmail.com; 9 Institute for Preventive Medicine and Public Health, Hanoi Medical University, Hanoi 100000, Vietnam; vohoanglonghmu@gmail.com (H.-L.V.); lan19yhdp@gmail.com (T.-L.N.); 10 National Hospital of Tropical Diseases, Hanoi 100000, Vietnam; phamngocthachnhtd@gmail.com (N.-T.P.); ntkhanhdp@gmail.com (T.-K.N.); 11 Ha Dong General Hospital, Hanoi 100000, Vietnam; dr.chinh68hd@gmail.com (D.-C.C.); vutrungy2e@gmail.com (V.-T.N.); 12 Dong Da General Hospital, Hanoi 100000, Vietnam; hoasythanoi@gmail.com (T.-M.T.T.); phambahien.bvdd@gmail.com (B.-H.P.); 13 Duc Giang General Hospital, Hanoi 100000, Vietnam; trananhlong64@gmail.com (A.-L.T.); thuongnhixanhpon@gmail.com (V.-T.N.); 14 North Thang Long Hospital, Hanoi 100000, Vietnam; bsnguyenthanhbvbtl@gmail.com (V.-T.N.); xuanthangbvbtl@gmail.com (X.-T.T.); 15 Disease Control and Health Emergency Program, World Health Organization Vietnam Country Office, Hanoi 100000, Vietnam; laiD@who.int (D.-T.L.); vuh@who.int (Q.-H.V.); otsus@who.int (S.O.)

**Keywords:** fear, psychophysiology, mental health, wellbeing, COVID-19

## Abstract

(1) Background: The present study measures the fear of COVID-19 among hospital healthcare workers and identifies several factors associated with increasing fear of COVID-19. (2) Methods: A cross-sectional, hospital-based survey was conducted on healthcare workforce recruited from the National Hospital of Tropical Diseases from 1 October 2021 and 20 October 2021. We selected the participants who have been directly involved in diagnosing, treating, or providing nursing care to patients with COVID-19. The primary data was collected via sending the invitation directly to the participants, utilizing structured self-completed questionnaires. The seven-item fear of COVID-19 scale was used to measure the data. The responses of 208 hospital healthcare workers were included in the final analysis. (3) Results: Total score of COVID-19 fear was 19.62 (SD = 5.22). The COVID-19 fear score of 7 items ranged from 2.38 (SD = 0.83) to 3.21 (SD = 0.96). The lowest and highest scores were the item ‘*My hands become clammy when I think about Corona*’ and the item ‘*I am most afraid of corona*’ was the highest, respectively. Linear regression of the COVID-19 fear showed that the factors positively correlated with the fear of COVID-19 among hospital healthcare workers were: being influenced by the community (*p* = 0.001), feeling at very high risk of COVID-19 (*p* = 0.03), and experiencing traumatic stress with an academic event (*p* = 0.042). (4) Conclusions: Although these findings merit further elaboration, these preliminary findings suggest relatively great fear of the COVID-19 pandemic among Vietnamese hospital healthcare workers and that social and personal connections are necessary for maintaining the mental wellbeing.

## 1. Introduction

Under the urgent effects of the coronavirus disease 2019 (COVID-19) pandemic on all facets of humanity, the fear of COVID-19, an emotional response to a threat by COVID-19 related issues, has quickly gained attention among different populations. Development and validation for the fear of COVID-19 scale were first developed by Daniel Kwasi Ahorsu et al. among the general Iranian population, which was reported in the early stages of the global COVID-19 pandemic [1]. Then, a series of researches using the fear of COVID-19 scale have been reported [2,3]. There was high fear of COVID-19 overall around the world, and it was more common among the female population and the highest in Asia; therefore, it seems necessary to pay more attention to the adverse impact of the COVID-19 pandemic on psychological issues.

As the COVID-19 pandemic escalates, measuring fear of COVID-19 in hospital healthcare workforce has an important role in understating the mental burdens placed on them. Beside the constant fear of every individual, people may not think clearly and rationally when responding to COVID-19. When fear is understood as a negative emotion involved in the processing of threat signals to the individual with an elevated level under the circumstance with stimuli [4,5], herein fear of healthcare workers arises with the threat of being harmed by the COVID-19 pandemic in terms of the risk of infection, and the pressures of work, family, and society. Consequently, greater fear associated with various psychological outcomes and worsening pre-existing psychiatric disorders has been shown in psychological studies during the COVID-19 pandemic [6,7]. Although fear of COVID-19 has been reported to be relatively high among healthcare professionals in general [2], we would like to have a more complete understanding of fear among healthcare professionals who are directly involved in the treatment of COVID-19 patients in medical isolation zones. This workforce was considered as leading healthcare professionals who played an indispensable role during the patient’s recovery as well as in the management of emergency patients in serious condition with confirmed or suspected COVID-19. Because of their close contact daily in isolation treatment zones, this workforce are at very high risk of catching the virus from COVID-19 patients and certain people could also become an important cause of virus transmission [8,9,10,11]. Since the onset of the COVID-19 in November 2019, there have been a huge number of studies navigating the effects of the pandemic on mental health among various healthcare workers. However, we hypothesize whether the fear level of hospital health workers who have directly involved in the diagnosis, treatment, and care of patients with COVID-19 is higher than that of the general population as well as other healthcare workforce. Since the onset of the COVID-19 pandemic, Vietnam has experienced four waves of SARS-CoV-2 infection with 905,477 confirmed COVID-19 cases and an average of approximately 9.193 cases per million people. In particular, during the fourth wave of COVID-19 since 27 April 2021, 900,669 confirmed cases have been reported nationwide, including 21,910 deaths [12]. When the number of COVID-19 infections in Vietnam reached its peak during the fourth wave, and a nationwide stay-at-home order had been issued, the current study was conducted to measure the fear of COVID-19 among hospital healthcare workers and to identify several potential factors associated with increasing fear of COVID-19. Findings of this study will provide inputs for policymakers and healthcare worker administrators on how to effectively support the mental health of frontline hospital healthcare workers in medical isolation areas of the COVID-19 treatment and sustain a well-engaged hospital healthcare workforce, particularly during this time of pandemic.

## 2. Methods

### 2.1. Study Design and Participants

The study is a cross-sectional, hospital-based survey carried out on healthcare workers who were recruited from the National Hospital of Tropical Diseases (base 2, Hanoi, Vietnam) from 1 October 2021 and 20 October 2021. A convenience sampling method was used in this study. Eligibility criteria included the participants (1) aged ≥18 years; (2) being hospital healthcare workers who obtained a contract to work full-time or part-time at the hospitals including medical doctors, nurses, midwives, and technicians; (3) having been providing direct medical care (diagnosing, treating or nursing care) to patients with COVID-19, and (4) who agreed to participate in the survey by providing an informed consent. We did not include healthcare workforce individuals who were not taking care of COVID-19 patients from the outpatient clinics.

A sample of 20 respondents was chosen for the pilot study to test its validity. The primary data was collected via sending the invitation directly to the participants, utilizing structured self-completed questionnaires in Vietnamese version. No material incentives were suggested to the respondents for their engagement of the survey to prevent them from answering more than once. The final analysis did not include the data from the pilot survey. In total, the responses of 208 hospital healthcare workers were included in the final analysis between 1 October 2021 and 20 October 2021.

### 2.2. Study Measurements

The study questionnaire was developed by a group of psychiatrists from the National Institute of Mental Health (Hanoi, Vietnam) and public health experts from the Hanoi Medical University (Hanoi, Vietnam) to collect the potential data on profession-related and socio-demographic characteristics, psychological trauma-related in the past one week, COVID-19 control and prevention-related characteristics, and psychological status of hospital healthcare workers.

The fear of COVID-19 scale, a seven-item scale that was developed using both a classical test theory and a Rasch analysis based on the general Iranian population, aimed to assess specific anxieties regarding COVID-19 [1]. The seven items *(*“*I am most afraid of coronavirus-19*”; “*It makes me uncomfortable to think about corona*”; “*My hands become clammy when I think about Corona*”; “*I am afraid of losing my life because of Corona*”; “*When I watch news and stories about Corona on social media, I become nervous or anxious*”; “*I cannot sleep because I’m worrying about getting Corona*”; “*My heart races or palpitates when I think about getting Corona*”*)* were rated with a five-item Likert point response (1: *strongly disagree*—5: *strongly agree*) and its total scores ranged from 7 to 35. The higher the score, the greater the fear of COVID-19. For the Vietnamese fear of COVID-19 scale (https://doi.org/10.31219/osf.io/v3n4u, accessed on 15 September 2021), the items were independently translated by a mother-tongue translator and internationally accepted practices for translation were employed [13]. The validated scale of COVID-19 fear was reported with good reliability in various medical settings in Vietnam (Cronbach’s α = 0.90–0.92) [14,15]. The Cronbach’s α of fear of COVID-19 scale in this study was 0.87. Additionally, the Vietnamese fear of COVID-19 scale was piloted on 20 participants who were known as physicians and nurses in Bach Mai Hospital of different ages, to investigate if there were any problems in understanding the items themselves. To avoid the effect of the order and the sequence, we presented the random order of the items of fear of COVID-19 scale for those 20 participants in the pilot survey.

### 2.3. Study Variables

#### 2.3.1. Main Outcome Variables

The main study outcome variable (or dependent variable) was presented as quantitative total score after calculating according to the fear of COVID-19 scale.

#### 2.3.2. Independent Variables

*Profession-related and socio-demographic variables* included age (years), gender (male, female), marital status (married, single, widowed/divorced), number of people living with (people), family household with own children under 18 years (no, yes), family household with own older person above 60 years (no, yes), education (lower secondary/upper secondary, college, university, postgraduation), profession (medical doctor, nurse and midwife, others), medical specialty (internal medicine, surgery, infectious disease, resuscitation and emergency medicine, anesthesiology, others), alcohol (no, yes), smoking (no, yes), comorbidities (no, yes), and using pain relief medications (no, yes).

*Psychological trauma-related variables* among hospital health workers: participants were asked whether they had experienced traumatic stress in the past one week of the aspects including family, work, academic, social, disease, and economic events (no, yes).

*COVID-19 control and prevention-related variables* included the severity of COVID-19 patients who were treated (normal level, mild level, moderate level, severe level), duration participating in COVID-19 control (months), knowledge preparation before participating in COVID-19 (no, yes), full equipment in current workplace conditions (no, yes), being affected by workplace conditions (no, yes), being affected by the community (no, yes), the feeling with COVID-19 infection risk (no, yes), and having a relative/friend/colleague with positive COVID-19 (no, yes).

### 2.4. Data Analysis

The data obtained were entered in EpiData 3.1 (The EpiData Association, Odense, Denmark), and the responses were coded appropriately before being exported to Stata^®^ 15 (StataCorp LLC, College Station, TX, USA) for analysis. A descriptive statistical analysis was first used to characterize the samples of hospital healthcare workers by work-related and socio-demographic variables, COVID-19 control and prevention work-related characteristics, and psychological trauma-related characteristics. Frequencies and proportions for each categorical variable were calculated and described, while quantitative variables were expressed as mean, standard deviation (SD) and interquartile range (IQR). Second, frequencies and proportions were used for seven psychometric properties of the fear of COVID-19 scale, then seven items and total score of COVID-19 fear were calculated with mean, SD, and IQR. To describe further the COVID-19 fear, we estimated the total score of COVID-19 fear according to selected study characteristics of hospital health workers, which was presented with mean, standard error, and 95% confidence interval (95% CI). Besides, the t-test and ANOVA test were applied to compare the differences of total score of COVID-19 fear among groups. Third, we employed the multiple linear regression model to analyse the association between included study variables and the COVID-19 fear. In this analysis, the total score of COVID-19 fear was treated as the outcome, while age, gender, marital status, number of people living with, family household with own children under 18 years, family household with own older person above 60 years, education, profession, medical specialty, alcohol, smoking, comorbidities, and using pain relief medications, experiencing traumatic stress in the past one week of the aspects including family, work, academic, social, disease, and economic events, the severity of COVID-19 patients who were treated, duration participating in COVID-19 control, knowledge preparation before participating in COVID-19, full equipment in current workplace conditions, being affected by workplace conditions, being affected by the community, the feeling with COVID-19 infection risk, and having a relative/friend/colleague with positive COVID-19 as independent variables. All possible predicting variables were included on the basis of psychiatry judgment and literature review. A *p*-value < 0.05 was considered statistically significant.

## 3. Results

### 3.1. Profession-Related, Socio-Demographic, and COVID-19 Control and Prevention Work Characteristics of Hospital Health Workers

As shown in Table 1, the mean age of the study sample was 33.20 (6.77) years; 62.02% of the participants were female. The majority of the medical staff was married (75.00%), while these figures for single and widowed/divorced were 20.19% and 4.81%, respectively. The proportion of those having own children under 18 years and own older person above 60 years were 67.31% and 27.40% each. Most of them graduated from college/university or higher (76.44%). More than half of the participants were nurses and midwives (57.69%), 27.40% were medical doctors, and 14.90% were medical technicians. Compared to other medical specialty, the percentage of participants who specialized in the infectious disease was the highest (28.37%). The proportion of healthcare workers who reported alcohol intake and smoking were 50.00% and 13.94%, respectively. Comorbidities were present in 48.08% healthcare workers and 2.88% of them had used pain relief medications.

**Figure 1:**
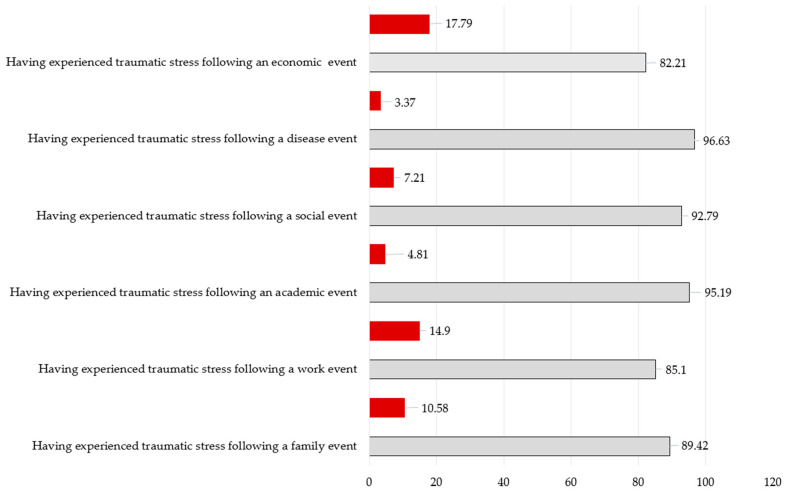
Psychological trauma-related characteristics in the past one week. Calculated as the percentage; red = YES; gray = NO.

Regarding COVID-19 control and prevention work-related characteristics, a total of 61 out of 208 healthcare workers (29.33%) participated in the treatment of severe COVID-19 patients, and 74 (35.58%) were involved in the treatment of moderate patients. Most healthcare workers participated in controlling COVID-19 (month) over one month (85.10%). The majority of healthcare workers had prepared knowledge before participating in COVID-19 (94.23%), and reported with full equipment in the current workplace conditions (93.75%). There were 38.46% healthcare workers affected by workplace conditions, and 37.98% were influenced a lot by the community. The feeling with high and very high risk from COVID-19 was common in participants (62.98%); 52.88% of healthcare workers had a relative/friend/colleague with positive COVID-19 (Table 1). As shown in Figure 1, the most common traumatic stress in medical staffs was following an economic event (17.79%) and a family event (10.58%).

### 3.2. Measurement of the COVID-19 Fear

Table 2 presents psychometric properties of the fear of COVID-19 scale in detail. The COVID-19 fear score of 7 items ranged from 2.38 (SD = 0.83) to 3.21 (SD = 0.96). Among them, the item ‘*My hands become clammy when I think about Corona*’ had the lowest COVID-19 fear score, and the score of the item ‘*I am most afraid of corona*’ was the highest. The total score of COVID-19 fear was 19.62 (SD = 5.22).

As presented in Table 3, there were significant differences of COVID-19 fear score by education (*p* = 0.034) and knowledge preparation before participating in COVID-19 (*p* = 0.0213). Compared to those without knowledge preparation before participating in COVID-19, the COVID-19 fear score was higher in those having knowledge preparation.

### 3.3. Associated Factors with the COVID-19 Fear among Hospital Healthcare Workers

In the linear regression analysis of the COVID-19 fear among hospital healthcare workers (Table 4), the factors positively correlated with the fear of COVID-19 among hospital healthcare workers were: being influenced by the community (*p* = 0.001), feeling at very high risk of COVID-19 (*p* = 0.03), and experiencing traumatic stress with an academic event (*p* = 0.042).

## 4. Discussion

Despite the recognition that COVID-19 fear and its consequence of psychological stress represent a major public health problem worldwide in this pandemic [16,17], research on COVID-19 fear in developing countries—where with inadequate vaccination rate and occurrence of SARS-CoV-2 Delta variant—is still lacking. To our knowledge, the present study is the first prospective documentation of the measurement of the COVID-19 fear among hospital healthcare workers who are directly involved in the treatment of COVID-19 patients in medical isolation zones in Vietnam. We have shown that the total score of COVID-19 fear was 19.62, which was higher than the pooled mean score (18.57) in recent systematic review reports from 44 articles [2], and was consistent with the mean of fear of COVID-19 that has been found in hospital staff in general (19.51) [2]. Among those, the item ‘*I am most afraid of corona*’ of the scale was the highest, consistent with previous reports [2].

In many of the studies, healthcare workers reported about experiencing stigma as a result of working on the pandemic [18]. This issue was the most common in the earlier phases of the outbreaks or in contexts where less was understood regarding virus transmission [18]. In particular, stigma is usually seen by the community around the individuals. Our study identified that hospital healthcare workers who reported being influenced by the community were more fearful than those who were not. Here, the authors have several arguments to argue for this relationship. First, media outlets including rumors and untruths are being flooded in the community with all kinds of COVID-19 information. With access to real-time information in this pandemic crisis, unverified information and a spike in social media rumors have somehow create a new breed of fear [19,20,21]. In addition to the wide and common role of the press and social media in spreading the Vietnamese government’s information related to COVID-19 pandemic in delivering risk communication messages to the public, the fake news phenomenon has been emerging substantially since the crisis impact of the COVID-19 pandemic [22]. Therefore, hospital healthcare workers being influenced by the community can also be understood as the exposure to negative risk communication. Second, while the uncertainty of the human about the future is beside the presence of this fear, a variety of anxiety aspects are formed in the human emotional system. Anxiety is the state of psychological, physiological, and behavioral emotion induced by a threat to well-being or survival [23,24], especially when a link was detected between fear of COVID-19 and anxiety [25].

Another notable finding from this study was the greater perceived fear in those having the feeling with very high COVID-19 infection risk. This finding was reasonable when previous evidence showed that healthcare workers who directly diagnosed, treated, or took care of COVID-19 patients were more stressed and psychologically impacted than those who were not involved in the direct treatment of COVID-19 [26]. Frontline healthcare professionals who are challenged in the diagnosis, treatment, and care of people who have suffered from COVID-19 experienced prolonged stress during this pandemic, as they were at high potential risk of COVID-19 infection due to the illness’ characteristics of high transmission efficiency, rapid deterioration, and pathogenicity. Hence, healthcare professionals, such as those having direct contact with infected patients, might suffer higher fear scores than those who were at low COVID-19 infection risk. Additionally, in the current study, a hospital healthcare worker who had documented traumatic stress with an academic event was associated with greater fear of COVID-19. This finding can be explained by the fact that in the group that engages directly with patients here, a significant number of young doctors are still in the process of studying graduate courses as well as further academical training programs. This is a personal matter to medical staffs which has been underrecognized and unaddressed in other areas and regions in previous studies. Therefore, in addition to the daily treatment of COVID-19 patients, they also have to take care of personal issues such as academic work.

The present findings should be interpreted within a context of potential weaknesses. Firstly, the seven-item Fear of COVID-19 Scale has not been validated in the general population in Vietnam yet; therefore, it is needed to examine the stability of the scale over time. The participants in this study were sampled from a specific population of hospital healthcare workers who were directly involved in the treatment of COVID-19 patients in medical isolation zones; as a result, we are not able to compare the level of fear with the group of medical professionals elsewhere. The study was carried out only in the National Hospital of Tropical Diseases (base 2, Hanoi, Vietnam), limiting the generalizability to other COVID-19 treatment facilities and/or settings of Vietnam.

## 5. Conclusions

We found relatively great fear of the COVID-19 pandemic among Vietnamese hospital healthcare workers and that the greater fear of COVID-19 was associated with the individuals being influenced by the community, feeling at very high risk of COVID-19, and experiencing traumatic stress with an academic event. Although these findings merit further elaboration, these preliminary findings suggest that social and personal connections are extremely important for maintaining healthcare professionals’ mental wellbeing.

## Figures and Tables

**Table 1:** Profession-related, socio-demographic, COVID-19 control and prevention-related, and psychological trauma-related characteristics of hospital health workers.

Profile of Hospital Health Workers		*N* = 208	Percentage (%)
Profession-related and socio-demographic characteristics			
Age—*Mean*; *SD* (*IQR*)		33.20 (6.77)	22–60
Sex	Male	79	37.98
	Female	129	62.02
Marital status	Married	156	75.00
	Single	42	20.19
	Widowed/Divorced	10	4.81
Number of people living with (people)	1–3 people	43	20.67
	4–5 people	114	54.81
	>5 people	51	24.52
Family household with own children under 18 years	No	68	32.69
	Yes	140	67.31
Family household with own older person above 60 years	No	151	72.60
	Yes	57	27.40
Education	Lower secondary/upper secondary	10	4.81
	College	95	45.67
	University	64	30.77
	Postgraduation	39	18.75
Profession	Medical doctor	57	27.40
	Nurse and midwife	120	57.69
	Medical technician	31	14.90
Medical specialty	Internal medicine	16	7.69
	Surgery	24	11.54
	Infectious disease	59	28.37
	Resuscitation and emergency medicine	19	9.13
	Anesthesiology	11	5.29
	Others	79	37.98
Alcohol	No	104	50.00
	Yes	104	50.00
Smoking	No	179	86.06
	Yes	29	13.94
Comorbidities	No	108	51.92
	Yes	100	48.08
Using pain relief medications	No	202	97.12
	Yes	6	2.88
**COVID-19 control and prevention work-related characteristics**			
Disease severity in COVID-19 patients	Normal level	32	15.38
	Mild level	41	19.71
	Moderate level	74	35.58
	Severe level	61	29.33
Duration participating in COVID-19 control (months)	<1 month	31	14.90
	1–3 month(s)	62	29.81
	>3 months	115	55.29
Knowledge preparation before participating in COVID-19	No	12	5.77
	Yes	196	94.23
Full equipment in current workplace conditions	No	13	6.25
	Yes	195	93.75
Affected by workplace conditions	No	128	61.54
	Yes	80	38.46
Affected a lot by the community	No	129	62.02
	Yes	79	37.98
Feeling at risk for COVID-19	Low risk	22	10.58
	Average risk	55	26.44
	High risk	103	49.52
	Very high risk	28	13.46
Having a relative/friend/colleague with positive COVID-19	No	98	47.12
	Yes	110	52.88

SD: standard deviation; IQR: interquartile range.

**Table 2:** Psychometric properties of the fear of COVID-19 scale.

Item	*N* = 208	Percentage (%)
I am most afraid of Corona		
Strongly disagree	13	6.25
Disagree	24	11.54
Neutral	94	45.19
Agree	60	28.85
Strongly agree	17	8.17
It makes me uncomfortable to think about Corona		
Strongly disagree	16	7.69
Disagree	27	12.98
Neutral	95	45.67
Agree	63	30.29
Strongly agree	7	3.37
My hands become clammy when I think about Corona		
Strongly disagree	32	15.38
Disagree	77	37.02
Neutral	87	41.83
Agree	10	4.81
Strongly agree	2	0.96
I am afraid of losing my life because of Corona		
Strongly disagree	32	15.38
Disagree	56	26.92
Neutral	61	29.33
Agree	49	23.56
Strongly agree	10	4.81
When I watch news and stories about Corona on social media, I become nervous or anxious		
Strongly disagree	22	10.58
Disagree	27	12.98
Neutral	85	40.87
Agree	64	30.77
Strongly agree	10	4.81
I cannot sleep because I’m worrying about getting Corona		
Strongly disagree	34	16.35
Disagree	60	28.85
Neutral	89	42.79
Agree	21	10.1
Strongly agree	4	1.92
My heart races or palpitates when I think about getting Corona		
Strongly disagree	38	18.27
Disagree	48	23.08
Neutral	87	41.83
Agree	31	14.9
Strongly agree	4	1.92
	**Mean (SD)**	**IQR**
I am most afraid of corona	3.21 (0.96)	1-5
It makes me uncomfortable to think about corona	3.08 (0.93)	1-5
My hands become clammy when I think about Corona	2.38 (0.83)	1-5
I am afraid of losing my life because of Corona	2.75 (1.12)	1-5
When I watch news and stories about Corona on social media, I become nervous or anxious	3.06 (1.02)	1-5
I cannot sleep because I’m worrying about getting Corona	2.52 (0.94)	1-5
My heart races or palpitates when I think about getting Corona	2.59 (1.01)	1-5
**Total score of COVID-19 fear**	19.62 (5.22)	7-35

SD: standard deviation; IQR: Interquartile range.

**Table 3:** Measure of the fear of COVID-19 by selected study characteristics of hospital health workers.

Profession-Related and Socio-Demographic Characteristics	Mean (SE)	95% CI		*p*-Value
		Lower	Upper	
Sex				0.9826 ^T^
Male	18.64 (0.62)	17.40	19.89	
Female	20.21 (0.43)	19.35	21.07	
Marital status				0.657 ^A^
Married	19.44 (0.42)	18.60	20.27	
Single	20.00 (0.80)	18.36	21.63	
Widowed/Divorced	20.80 (1.31)	17.82	23.77	
Number of people living with (people)				0.487 ^A^
1–3 people	20.16 (0.75)	18.64	21.67	
4–5 people	19.55 (0.51)	18.53	20.57	
>5 people	19.31 (0.67)	17.94	20.67	
Family household with own children under 18 years				0.6129 ^T^
No	19.47 (0.68)	18.09	20.84	
Yes	19.69 (0.42)	18.85	20.52	
Family household with own older person above 60 years				0.1609 ^T^
No	19.84 (0.42)	19.00	20.67	
Yes	19.03 (0.69)	17.64	20.42	
Education				0.034 ^A,^*
Lower secondary/upper secondary	19.10 (2.63)	13.13	25.06	
College	20.44 (0.48)	19.48	21.40	
University	20.48 (0.56)	19.34	21.62	
Postgraduation	16.33 (0.85)	14.59	18.06	
Profession				0.519 ^A^
Medical doctor	17.78 (0.68)	16.42	19.15	
Nurse and midwife	20.67 (0.44)	19.79	21.55	
Medical technician	18.90 (1.02)	16.80	21.00	
Medical specialty				0.271 ^A^
Internal medicine	20.50 (0.87)	18.63	22.36	
Surgery	18.12 (0.99)	16.07	20.17	
Infectious disease	18.20 (0.69)	16.82	19.58	
Resuscitation and emergency medicine	20.89 (1.19)	18.38	23.40	
Anesthesiology	21.81 (1.08)	19.39	24.23	
Others	20.34 (0.61)	19.10	21.57	
Alcohol				0.2332 ^T^
No	19.88 (0.50)	18.88	20.88	
Yes	19.35 (0.52)	18.32	20.38	
Smoking				0.2340 ^T^
No	19.72 (0.38)	18.95	20.49	
Yes	18.96 (0.99)	16.92	21.01	
Comorbidities				0.4159 ^T^
No	19.69 (0.46)	18.76	20.62	
Yes	19.54 (0.56)	18.42	20.65	
Using pain relief medications				0.8709 ^T^
No	19.54 (0.36)	18.82	20.27	
Yes	22.00 (1.80)	17.35	26.64	
**COVID-19 control and prevention-related characteristics**				
Disease severity in COVID-19 patients				0.062 ^A^
Normal level	19.59 (1.12)	17.29	21.89	
Mild level	19.31 (0.74)	17.81	20.82	
Moderate level	19.36 (0.52)	18.32	20.40	
Severe level	20.14 (0.73)	18.68	21.61	
Duration participating in COVID-19 control (months)				0.811 ^A^
<1 month	20.12 (1.00)	18.07	22.17	
1–3 month(s)	19.35 (0.64)	18.07	20.63	
>3 months	19.62 (0.48)	18.65	20.59	
Knowledge preparation before participating in COVID-19				0.0213 ^T,^*
No	22.58 (1.28)	19.76	25.40	
Yes	19.43 (0.37)	18.70	20.17	
Full equipment in current workplace conditions				0.0628 ^A^
No	21.76 (1.45)	18.58	24.94	
Yes	19.47 (0.37)	18.74	20.21	
Affected by workplace conditions				0.7720 ^T^
No	19.40 (0.42)	18.56	20.24	
Yes	19.96 (0.65)	18.65	21.26	
Affected a lot by the community				0.9999 ^T^
No	18.58 (0.46)	17.67	19.49	
Yes	21.31 (0.53)	20.24	22.38	
Feeling at risk for COVID-19				0.135 ^A^
Low risk	18.81 (1.29)	16.12	21.51	
Average risk	18.03 (0.60)	16.81	19.25	
High risk	20.32 (0.48)	19.35	21.28	
Very high risk	20.78 (1.17)	18.37	23.19	
Having a relative/friend/colleague with positive COVID-19				0.8420 ^T^
No	19.23 (0.51)	18.21	20.25	
Yes	19.96 (0.50)	18.95	20.97	
**Psychological trauma-related characteristics in the past one week**				
Having experienced traumatic stress following a family event				0.9308 ^T^
No	19.43 (0.37)	18.68	20.18	
Yes	21.18 (1.17)	18.73	23.62	
Having experienced traumatic stress following a work event				0.7574 ^T^
No	19.51 (0.37)	18.78	20.24	
Yes	20.22 (1.19)	17.78	22.66	
Having experienced traumatic stress following an academic event				0.2639 ^T^
No	19.67 (0.36)	18.94	20.39	
Yes	18.60 (1.98)	14.09	23.10	
Having experienced traumatic stress following a social event				0.9222 ^T^
No	19.47 (0.36)	18.75	20.19	
Yes	21.46 (1.73)	17.75	25.18	
Having experienced traumatic stress following a disease event				0.3208 ^T^
No	19.65 (0.36)	18.92	20.37	
Yes	18.71 (2.42)	12.77	24.65	
Having experienced traumatic stress following an economic event				0.8072 ^T^
No	19.47 (0.37)	18.72	20.22	
Yes	20.29 (1.04)	18.17	22.42	

^T^: *t*-test; ^A^: ANOVA Test; *: significant at 0.05; SE: standard error; 95% CI: 95% confidence interval.

**Table 4:** Associated factors with the COVID-19 fear among hospital healthcare workers: Multiple linear regression analysis.

Selected Variables	Coef.	SE	*p*-Value	95% CI	
				Lower	Upper
Age	0.04	0.07	0.500	−0.09	0.18
Gender (vs. Male)					
Female	0.83	0.98	0.394	−1.09	2.77
Marital status (vs. Married)					
Single	0.33	1.12	0.769	−1.89	2.55
Widowed/Divorced	0.99	1.78	0.578	−2.52	4.51
Number of people living with (vs. 1–3 people)					
4–5 people	−0.19	1.03	0.848	−2.23	1.83
>5 people	−1.02	1.26	0.419	−3.52	1.47
Family household with own children under 18 years (vs. No)					
Yes	1.41	0.87	0.107	−0.30	3.12
Family household with own older person above 60 years (vs. No)					
Yes	−1.38	0.89	0.124	−3.15	0.38
Education (vs. Lower secondary/upper secondary)					
College	0.67	2.26	0.766	−3.79	5.14
University	1.35	2.17	0.536	−2.95	5.65
Postgraduation	−2.63	2.44	0.282	−7.46	2.19
Profession (vs. Medical doctor)					
Nurse and midwife	−0.01	1.37	0.99	−2.72	2.68
Medical technician	−1.98	1.71	0.25	−5.37	1.40
Medical specialty (vs. Internal medicine)					
Surgery	−1.49	1.67	0.371	−4.80	1.80
Infectious disease	−1.23	1.54	0.426	−4.29	1.81
Resuscitation and emergency medicine	1.12	1.85	0.545	−2.53	4.79
Anesthesiology	1.89	2.13	0.377	−2.32	6.10
Others	0.98	1.47	0.506	−1.93	3.90
Smoking (vs. No)					
Yes	0.03	1.11	0.978	−2.17	2.23
Alcohol (vs. No)					
Yes	0.85	0.89	0.337	−0.89	2.61
Morbidity (vs. No)					
Yes	−0.04	0.85	0.954	−1.74	1.64
Using pain relief medications (vs. No)					
Yes	1.04	2.22	0.638	−3.33	5.43
Disease severity in COVID-19 patients (vs. Normal level)					
Mild level	−0.68	1.38	0.623	−3.41	2.04
Moderate level	−0.24	1.22	0.842	−2.65	2.16
Severe level	−0.55	1.33	0.676	−3.19	2.07
Duration participating in COVID-19 control (vs. < 1 month)					
1–3 month (s)	0.22	1.21	0.851	−2.16	2.62
>3 months	0.60	1.24	0.627	−1.84	3.05
Knowledge preparation before participating in COVID-19 (vs. No)					
Yes	−3.00	1.77	0.092	−6.51	0.50
Full equipment in current workplace conditions (vs. No)					
Yes	−3.14	1.69	0.065	−6.48	0.19
Affected by workplace conditions (vs. No)					
Yes	−0.24	0.82	0.771	−1.87	1.39
Affected a lot by the community (vs. No)					
Yes	2.71	0.82	0.001 **	1.07	4.35
Feeling at risk for COVID-19 (vs. Low risk)					
Average risk	0.48	1.41	0.729	−2.29	3.27
High risk	2.42	1.31	0.067	−0.16	5.01
Very high risk	3.63	1.66	0.03 *	0.34	6.92
Having a relative/friend/colleague with positive COVID-19 (vs. No)					
Yes	−0.0001	0.83	1.000	−1.63	1.63
Having experienced traumatic stress following a family event (vs. No)					
Yes	1.87	1.83	0.309	−1.74	5.49
Having experienced traumatic stress following a work event (vs. No)					
Yes	−2.06	1.61	0.202	−5.25	1.12
Having experienced traumatic stress following an academic event (vs. No)					
Yes	−4.62	2.25	0.042 *	−9.08	−0.17
Having experienced traumatic stress following a social event (vs. No)					
Yes	3.70	1.98	0.064	−0.21	7.61
Having experienced traumatic stress following a disease event (vs. No)					
Yes	−2.01	2.74	0.464	−7.42	3.40
Having experienced traumatic stress following an economic event (vs. No)					
Yes	0.06	1.36	0.96	−2.63	2.77
Pseudo R2	0.3374				

Coef.: coefficient; SE: standard error; 95%CI: 95% confidence interval; *, **: significant at 0.05 and 0.01, respectively.

## Data Availability

The data used to support the findings of this study are available from the corresponding author upon request.
